# Perceived oral health and its association with symptoms of psychological distress, oral status and socio-demographic characteristics among elderly in Norway

**DOI:** 10.1186/s12903-018-0556-9

**Published:** 2018-05-31

**Authors:** Kari Elisabeth Dahl, Giovanna Calogiuri, Birgitta Jönsson

**Affiliations:** 1 Department of Public Health, Faculty of Social and Health Sciences, Inland Norway University of Applied Sciences, Hamarveien 112, 2418 Elverum, Norway; 2 The Public Dental Health Service. Competence Centre of Northern Norway (TkNN), Tromsø, Norway; 3 Institute of Odontology, Department of Periodontology, The Sahlgrenska Academy, University of Gothenburg, Gothenburg, Sweden

**Keywords:** Ageing, Mental health, Depression, Older adults, Epidemiology

## Abstract

**Background:**

There is poor knowledge about the extent to which psychological distress influences oral health in older people in Norway. The aim of this study was two-fold: i) to describe the oral health of Norwegian elderly and their levels of psychological distress; and ii) to examine the relationship of psychological distress with self-rated oral health, while controlling for oral status and socio-demographic characteristics, in Norwegian elderly.

**Methods:**

Data were retrieved from a national cross-sectional survey conducted by Statistics Norway in 2012 and included information about self-rated oral health, psychological distress (measured using the Hopkins Symptom Checklist 25; HSCL-25), gender, age, civil status, smoking, self-reported number of teeth present and dental attendance for 949 non-institutionalised adults aged 65 years or older. Logistic regression was used to establish whether psychological distress predicts self-rated oral health, controlling for socio-demographic characteristics and oral status.

**Results:**

Around 27% of the elderly reported having poor oral health, and 8 % had a HSCL-25 mean score ≥ 1.75, which indicates higher levels of psychological distress. Among the symptoms listed in the HSCL-25, the most frequently reported problems were lack of energy (1.7 ± 0.8) and difficulties falling and staying asleep (1.6 ± 0.7). The likelihood of reporting poor oral health was independently associated with having a mean HSCL-25 score ≥ 1.75 (OR = 1.89; 95% CI = 1.14–3.15), even when smoking (OR = 1.83; 95% CI = 1.17, 2.87) and having fewer than 20 teeth (OR = 3.49; 95% CI = 2.56, 4.76) were taken into account.

**Conclusion:**

Most of the Norwegian elderly in our sample perceived themselves to have good oral health and reported relatively low levels of psychological distress. Higher levels of psychological distress can influence the oral health of the elderly independently of other factors such as smoking and having reduced number of teeth. Dental care professionals should consider screening their elderly patients for psychological distress and individualise the information about dental care for this specific population.

## Background

As in other European countries, in Norway an increasing decline in fertility rates and increased life expectancy are resulting in an increase of the proportion of older people which represent a potential burden to society and a challenge to public health institutions [1](WHO). Compared with other European countries, only Poland, Iceland and Ireland have fewer elderly people than Norway [2]. However the population age is increasing at varying rates. For example, the proportion of the Norwegian population aged 65 years and older in 2015 was 16.3%, an increase from 8.4% in 2006. In order to ensure people’s quality of life and to minimise increased public costs, it is important to understand how to promote good health among the increasing number of older citizens.

One challenge in becoming elderly is maintaining good oral health, especially for fragile or cognitively impaired older persons, which is one of the reasons why elderly people have received increasing attention from researchers and health policy makers [3]. To maintain good oral health is, an important component of a healthy ageing [4]. A number of dental conditions are associated with older age, such as dry mouth (xerostomia), root caries, and periodontal disease [5–7]. Periodontitis, which may result in tooth loss, is still a common disease and its prevalence is reported by European and US studies to range from 31% to 76% [8–10]. Furthermore, its prevalence and severity increase with age [6, 7]. During recent decades, a reduction in the prevalence of edentulousness and the prevalence and incidence of tooth loss has occurred in many countries [11, 12]. Despite these observed declining trends in edentulousness, the mean number of lost teeth increases with increasing age and a substantial proportion of the current older generations experience tooth loss [11, 13]. Furthermore, studies have confirmed the expected positive associations between tooth loss and reduced perceived oral health [13, 14].

While most of the elderly have good mental health and psychological well-being, many are at increased risk of developing mental disorders [15]. Symptoms of psychological distress are common and serious, causing significant disruption in daily living. Mental disorders account for circa 7.4% of the global burden of disease and will probably steadily increase in the future [16]. Psychological distress is often under-recognised and under-treated in primary care [17]. Psychological distress is so widespread that it can be considered one of the most common health problems in Norway [18]. It is estimated that about 30% of the Norwegian population experience high levels of psychological distress at some time during their lifetime [18]. Psychological distress is highly prevalent in the elderly and often has negative consequences on their everyday life, such as leading to a reduced quality of life [19]. Increased psychological distress might also have a negative impact on the elderly‘s health-related behaviours, including their dietary patterns as well as their dental hygiene behaviours, which can in turn lead to poor oral health [20]. Symptoms of psychological distress have been found to be associated with tooth loss and use of dental care services in the U.S. [21] and in a Finnish populations [22, 23]. However, in Norway, knowledge about how non-institutionalised older adults perceive their oral health and how often they have check-ups still remains scanty [24]. Furthermore, little is known about the extent to which psychological distress affects the oral health of Norwegian elderly [21, 24].

The aim of this study was two-fold: i) to describe Norwegian elderly’s self-rated oral health and levels of psychological distress; and ii) to examine the relationship between psychological distress and self-rated oral health while controlling for oral status, smoking, dental attendance and sociodemographic characteristics.

## Methods

### Study population

The data for this study were retrieved from a national Norwegian cross-sectional study conducted by Statistics Norway (SSB) in 2012: “The Living Conditions study – Health, health care and social contact” [25]. A representative random sample of 10,000 Norwegians aged 16 years and older was drawn, stratified by gender, age group and region of residence, from SSB’s demographic/population database BEREG, a database that is updated from The Central Population Register. Of the total drawn from the database, 229 persons had died or were living abroad, consequently, these were not invited to participate, giving a gross sample of 9771 (100% of the invited participants) persons of whom 4111 declined to participate (42%) giving a net samples of 5660 people (58%). For this study, only respondents aged 65 years and above were used for the analyses (*n* = 949).

The samples were balanced in respect of gender, age and region of residence where everyone in the household was considered as one unit [25].

### Data collection process

Persons in the study were invited to participate by a letter from the SSB, which provided information in Norwegian regarding the purpose of the study, the procedures and actions taken to ensure confidentiality and stating that they would be contacted by telephone for an interview. Data collection involved a combination of a phone interview and a follow-up self-administered questionnaire. Before the interview started, all respondents consented to participate. The follow-up questionnaire was sent to the participants two to three weeks after the phone interview and could be completed either on paper or on a web site.

### Instruments

Self-reported oral health, which was the dependent variable in the study, was assessed with one question in the follow-up questionnaire: “How do you perceive your dental health to be?” Five response options were provided: “Very poor”, “poor”, “neither poor nor good”, “good”, or “Very good”.

Psychological distress, which was the primary predictor in this study, was assessed using the Hopkins Symptoms Checklist − 25 (HSCL-25). HSCL-25 is a shortened version of an original 90 items questionnaire (HSCL-90) and is one of the most commonly used questionnaires to measure the prevalence of mild psychological distress in the population [26–29]. A Norwegian version of this instrument has been validated and used for research and clinical purposes since the late 1980s [30]. The scale consists in one question followed by 25 statements that describe symptoms of two major components of psychological distress, Anxiety and Depression: “Specify how much each problem has plagued you or caused trouble during the past 14 days? Each statement was rated by the participants on a four point Likert scale”, with each symptom being rated on a 4-point scale (1= “Not at all”, 2 = “A little”, 3= “Quite a bit”, and 4 = “Extremely”). The first ten items in the HSCL-25 questionnaire concern anxiety symptoms and the following 15 items concern depression symptoms. A total mean score ≥ 1.75 indicates psychological distress at a case level [31]. The overall internal consistency for the HSCL-25 was high (alpha = 0.95). All individual items of the HSCL-25 are presented in Table 1.

**Table 1:** Proportions of responses to each item on the Hopkins Symptom Checklist-25 (*n* = 949)

Item	Not at all	A little	Quite a bit	Extremely	Mean (SD)
1. Headache	73.9	22.1	2.6	0.5	1.3 (0.6)
2. Tremor	86.0	9.7	1.8	0.6	1.2 (0.4)
3. Lassitude or dizziness	66.2	27.9	4.1	0.5	1.4 (0.7)
4. Nervousness, restlessness	66.5	29.0	3.2	0.7	1.4 (0.6)
5. Suddenly scared for no reason	88.3	9.3	1.8	0.2	1.1 (0.4)
6. Increasingly fearful or anxious	85.7	11.1	2.2	0.1	1.2 (0.6)
7. Palpitations, heart beat running away	77.9	18.2	1.8	0.2	1.3 (06)
9. Bouts of anxiety or panic	91.7	6.5	0.8	0.2	1.1 (0.4)
10. So restless that it is difficult to sit still	84.7	12.9	1.3	0.5	1.2 (0.5)
11. Lack of energy, everything goes slower than usual	40.8	20.2	9.5	2.0	1.7 (0.8)
12. Blaming yourself for things	63.5	30.2	4.6	0.7	1.4 (0.7)
13. Tearfulness	76.0	20.0	2.8	0.7	1.3 (0.5)
14. Thoughts about taking your life	96.3	2.4	0.7	0	1.0 (0.2)
15. Poor appetite	90.1	7.9	1.3	0.3	1.1 (0.5)
16. Difficulty falling asleep, staying asleep	55.6	33.6	7,6	2.5	1.6 (0.7)
17. Sense of hopelessness about the future	74.4	22.1	2.4	0.6	1.3 (0.5)
18. Depressed, melancholy	78.4	18.4	1.8	0.4	1.2 (0.6)
19. Feeling of loneliness	74.3	21.1	3.5	0.5	1.3 (0.6)
20. Loss of sexual desire and interest	53.5	29.7	9.7	3.8	1.6 (0.8)
21. Feeling of being cheated in a trap or trapped	93.8	5.0	0.5	0.4	1.0 (0.3)
22. Very worried or upset	70.3	25.6	3.4	0.4	1.3 (0.6)
23. No interest in anything	87.2	10.5	1.4	0.4	1.1 (0.5)
24. Feeling everything is an effort	75.2	20.2	3.1	0.9	1.3 (0.6)
25. Feeling useless	79.3	17.1	2.6	0.9	1.2 (0.5)
Anxiety mean score					1.2 (0.3)
Depression mean score					1.3 (0.3)
HSCL-25 total mean score					1.3 (0.3)

When numbers in columns do not equal 100%, there is internal drop-out in the questionnaire

Other control variables in this study include oral status, smoking, dental attendance, and sociodemographic characteristics. Oral status was assessed with one variable: Number of teeth present. “Roughly, how many of your own teeth do you have left?” which was answered by choosing one of four response options: 1) “20 teeth or more”, 2) “10–19 teeth”, 3) “1–9 teeth”, and 4) “none”. Two variables assessed habits that could have an impact on dental status and oral health: Smoking habits and dental attendance. Smoking was assessed with the question: “Do you smoke?” to which the respondents answered “yes” or “no”. Dental attendance was assessed with the question: “When did you last visit a dentist?”, which were answered by choosing one of five response choices: 1) “6 month ago or less”, 2) “7-12 months ago”, 3) “1–2 years ago” 4) “more than two years but less than five years ago”, 5) “more than 5 years ago”.

Gender, Age group (65–74 years and 75+ years), and Civil Status (i.e. living with partner or Single) were used as selected sociodemographic characteristics.

### Statistical analysis

Descriptive statistics was used to address the first objective of our study. Self-rated oral health was dichotomised: 1) Good oral health (comprising the response alternatives “Very good” and “Good”), and 2) Poor oral health (comprising the response alternatives “Neither good nor poor”, “Poor” and “Very poor”). The individual items of the HSCL-25 and the Anxiety and Depression components are presented as means (M) and standard deviations (SD), while the total score of the HSCL-25 is presented as prevalence of individuals with mean score above or below the 1.75 cut-off, i.e. individuals with lower or higher levels of psychological distress [31].

In order to address the second objective of our study, logistic regressions were performed. Before performing the Logistic regression analysis, the responses to self-assessed number of teeth present were collapsed into two categories (20 teeth and more vs. 0–19 teeth) and dental attendance into two categories (within 6–12 months vs. more than 12 months). Those variables that were significant in the univariate analysis were included in a multivariable logistic regression analysis in order to examine the extent to which Psychological distress predicted Self-reported oral health while controlling for the respondents’ sociodemographic characteristics, oral health status, smoking habits and dental attendance. The associations are presented as odds ratios (ORs) and 95% confidence intervals (95% CI). The Hosmer-Lemeshow goodness-of-fit test was used to examine whether the final model adequately fitted the data. The significance was set at *p* > 0.05. All data analyses were performed using the Statistical Package for the Social Sciences (SPSS, Version 24.0, IBM Armonk, NY).

## Results

The participants’ ages ranged between 65 to 96 years, with the median age being 69 years (quartile deviation 68–73 years). The sample consisted of 54.4% (514) women and 45.6% (431) men. A total of 71.3% (*n* = 674) rated their oral health as good or very good and 28.7% (271) rated their oral health as very poor, poor, or neither good nor poor. There were no differences between men and women on how they rated their oral health (χ^2^ 0.093, df 1, *p* = 0.760) or between age groups (χ^2^ 0.130, df1, *p* = 0.718).

The individual HSCL-25 items are presented in Table 1. Missing values for each question varied from 0.2% to 3.3%, with the highest internal loss for the item concerning ‘Loss of sexual desire and interest’ (3.3%). For 23 of the 25 items, the missing values were under 1.0%.

In the overall sample, the total mean score of the HSCL-25 was 1.3 ± 0.32. The overall score for the Anxiety component was 1.2 ± 0.3, the mean score for the Depression component was 1.3 ± 0.3. Eight per cent of the participants had a total HSCL-25 mean score above the 1.75 cut-off, indicating higher levels of psychological distress, while the prevalence of those having a mean score above the 1.75 cut-off for Anxiety was 6.3% and Depression was 10.4%. Among the various symptoms listed in the HSCL-25, “Lack of energy” (1.7 ± 0.8), “Difficulties falling and staying asleep” (1.6 ± 0.7), and “Loss of sexual desire and interest” (1.6 ± 0.8) were rated with the highest mean scores. Women had significantly higher HSCL-25 mean scores than men (1.32 ± 0.33 and 1.26 ± 0.30, respectively; ANOVA: F = 7.46, *p* = 0.006), and the elderly in the oldest age group had significantly higher HSCL-25 mean scores than the youngest age group mean (1.36 ± 0.34 and 1.27 ± 0.30, respectively; ANOVA: F = 5.99, *p* = 0.003).

In Table 2, predictors of self-reported oral health are presented. Totals of 65.4% and 89.2% were living with partner and were non-smokers, respectively. Moreover, totals of 65.9% and 83.2% had 20 teeth or more and attended a dentist annually, respectively.

**Table 2:** Predictors of self-rated poor oral health among elderly Norwegian adults

Variables	Categories	Crude measures		Univariable			Multivariable^a^		
		N	Poor self-rated oral health %	OR	95% CI	*P*	OR	95% CI	*P*
HSCL-25 diagnose	Mean score > 1.75	75	45.3	2.21	1.37–3.57	0.001	1.89	1.14–3.15	0.014
	Mean score ≤ 1.75 (reference)	870	27.2	1.00			1.00		
Civil status	Single	327	33.9	1.47	1.09–1.97	0.009	1.16	0.85–1.59	0.345
	Living with partner (reference)	618	25.9	1.00			1.00		
Smoker	Yes	102	46.1	2.36	1.55–3.59	< 0.001	1.83	1.17–2.87	0.008
	No (reference)	843	26.6	1.00			1.00		
Number of teeth	0–19	322	47.2	3.79	2.82–5.09	< 0.001	3.49	2.56–4.76	< 0.001
	≥ 20 (reference)	623	19.1	1.00			1.00		
Dental attendance	> 12 month	153	35.3	1.48	1.02–2.13	0.037	0.93	0.62–1.40	0.746
	6–12 month (reference)	786	27.0	1.00			1.00		

OR = Odds ratio; 95% CI = 95% confidence Interval. ^a^Model adjusted for the interaction between HSCL-25 diagnose, civil status, smoking, number of teeth, and dental visits

In the univariable analysis, the likelihood of perceiving poor oral health was increased for individuals who had symptoms of psychological distress, lived alone, smoked, had fewer than 20 teeth, and for persons’ who had not attended the dentist within the past year.

When the model was adjusted for all variables in a multivariable model, the participants who had higher levels of psychological distress were 1.89 times more likely to report poor oral health, even when other factors were controlled. Smokers were 1.83 times more likely to perceive poor oral health than non-smokers and those with fewer than 20 teeth were 3.49-times more likely to perceive poor oral health than those with more teeth. In the final model, living alone and not having attended the dentist within the past year were not significantly associated with poor oral health. The goodness-of-fit of measurement used in the model was an acceptable fit on the Omnibus test of model coefficient (χ^2^ 92.98, *p* > 0.001) and Hosmer and Lemeshow test (χ^2^ 2.61, *p* = 0.760). The factors used as predictor in the model explained 13.5% of the variance of perceived poor oral health (Nagelkerke’s R^2^ 0.135).

## Discussion

The first objective of our study was to describe oral health and psychological distress among Norwegian elderly. The majority of the elderly in our sample were non-smokers, had 20 teeth or more, and had attended a dental check-up within the past 12 months. Nearly one-third of the elderly reported having poor oral health, and 8% had higher levels of psychological distress. Among the symptoms listed in the HSCL-25, the most frequently reported problems were lack of energy and difficulties falling and remaining asleep. There were no differences between men and women, or between age groups, in the extent to which they rated their oral health, though women and those in the oldest age group showed higher levels of psychological distress than men and those in the youngest age group, respectively. The second objective of our study was to examine the relation of psychological distress with perceived oral health, controlling for sociodemographic characteristics, smoking, oral status, and dental attendance. The likelihood of reporting poor oral health was independently associated with higher levels of psychological distress while controlling for the other independent variables. Smoking and, especially, having fewer than 20 teeth were also highly significant predictors of poor oral health.

Nearly 66% of the elderly in our sample had 20 teeth or more, confirming the high proportion of elderly people in Norway who retained their natural teeth, as shown in earlier studies [14, 32–34], which is considered essential to maintain adequate chewing function and normal oral health-related quality of life [35, 36].

In line with our findings, a study carried out in nine European countries showed that the occurrence of symptoms of depression in older people living at home varied between 8.0 and 23.6%, with a higher prevalence in women than in men [37]. Although we found the prevalence of Norwegian elderly with higher levels of psychological distress was somewhat low, symptoms such as lack of energy and difficulties falling and remaining asleep were commonly reported. Such symptoms might give an indication of mood and psychological distress and should therefore be regarded in light of their possible impact on the elderly’s oral health. Furthermore, the prevalence of psychological distress is likely to increase in future, alongside the increase in the ageing population, and this represents a challenge in Norway and other countries [38, 39].

Our findings relative to the association of psychological distress with oral health are consistent with the literature. Previous studies have in fact found an increased risk for impaired oral health among adults with anxiety and depressive symptoms [21, 22]. For example, in a Finnish study [23], elderly with symptoms of anxiety or depression were almost twice as likely to experience poor oral health. Women with high rates of depressive symptoms had more negative attitude toward preserving their natural teeth, used sugary products more frequently and did not attend for dental check-ups regularly, compared with non-depressed women. Further, depressive symptoms were associated with edentulousness among non-smoking men and symptoms of anxiety were significantly associated with lower tooth brushing frequency [21, 23]. Similarly, in a study on a U.S. population, Okoro et al. found that adults with psychological disorders such as depression and anxiety were less likely to attend oral health services and to have tooth loss or had one or more teeth removed than those without such disorder, after controlling for multiple confounders [21].

Higher levels of psychological distress are often associated with loss of energy and might result, for example, in less effective dental hygiene and difficulty following-up routines such as general dental care attendance. Dry mouth is a common complaint in older people, persons suffering from dryness of the mouth are likely to experience several oral problems, including high levels of caries, in addition to difficulties in chewing, eating and communicating [36]. The appetite often declines alongside interest in cooking meals. This can lead to more pronounced loss of energy due to lack of nutrition [20] and such persons show less interest in socialising with other people.

In our study, we also found that the highest adjusted odds ratios were observed for having fewer than 20 teeth, which is also in line with the literature [33, 40, 41]. Musacchio et al. [42] found that tooth loss impacts on general health and is a risk factor for malnutrition, disability, loss of self-sufficiency, and deterioration in quality of life, and having fewer teeth was associated with social lifestyle factors in a population of elderly Italians. Despite an increasing number of people retaining their natural teeth throughout life, the prevalence of oral diseases increases among the institutionalised elderly, their objective need for dental treatment is therefore even greater than before [38, 43].

Ekback et al. [39] showed that elderly persons missing many teeth were less likely to be satisfied with their oral condition than those missing only a few or no teeth. Individuals who perceived they had poor general health, smoked daily, had tooth loss, experienced toothache, had difficulties with chewing, and reported bad breath were less likely than their counterparts to be satisfied with their oral health status.

### Implications of our findings

Our findings underline the necessity for dental professionals to be aware of elderly people’s levels of psychological distress, as this can have a negative impact on oral health and oral-health related quality of life. Consequently, dental care professionals should consider screening their elderly patients for psychological distress. Moreover, the importance of oral care for older people with psychological distress needs to be emphasised by dental professionals, to reduce the possibility of poorer oral health and deterioration of quality of life in this population.

As tooth loss has a negative impact on how people perceive their oral health is essential in order to maintain adequate chewing function and higher standards of oral health related quality of life [36]. Regular oral health care check-ups are important for helping elderly people maintain their natural teeth through periods when they are in poor health [44, 45]. Therefore, it is important that systematic dental care services for older adults are accessible and affordable [45, 46].

### Strength and weaknesses of the present study

The HSCL 25 has been used to assess psychological distress in a number of age and cultural contexts [31, 47, 48]. The way in which the HSCL-25 questionnaires is administrate differs slightly. For example, statements can be delivered face-to-face by a trained interviewer or, like in the present study, the instrument can be self-administered. This may account for some differences in the overall HSCL scores, though evidences suggest that the method of delivery should not have a major impact on the total scores. Nonetheless, in view of this methodological difference, the main emphasis in this analysis has been on the patterns of association rather than absolute differences in the questionnaire scores. Our study is subject to several limitations. First, all data are self-reported. The HSCL-25 was however found to perform well in assessing the symptom severity of anxiety and depression distress among non-elderly in general, and it could therefore be considered as a suitable and valid measure for elderly people. A critical issue in the clinical application of HSCL-25 is to identify cut-off points on the scale that can be used to support decisions about whether further clinical examination is necessary. In our study, the 1.75 cut-off was applied, which has previously shown high validity in a Swedish population [31] and which can be expected to be fairly similar to Norwegian populations in terms of language and cultural context. Another limitation is that the participants’ oral status (number of remaining teeth) was self-reported and thus might not correspond to the actual remaining number of natural teeth. However, previous studies have shown a close agreement between self-reported and clinically measured dentition status. [49, 50]. The use of only clinical measures to assess oral health of individuals has been criticized because they fail to consider functional and psychosocial aspects of health and do not adequately reflect the functioning, concerns and perceived needs of individuals [51, 52]. It is hereby of interest also to include patients` assessment of their wellbeing in the term oral health. In addition, there is a growing interest in dentistry also to assess the influence of oral health on daily life, often labelled oral health-related quality of life (OHRQoL) [52, 53].

Furthermore, tooth loss is considered as an effective marker of the population oral situation. [54]. One of the major limitation of this study relates to possible self-selection bias. The participants in this study were probably healthy elderly living at home, able to manage themselves in everyday life, while the ill or less independent elderly who were unable or did not wish to participate might have affected the responses and the results. In a study by Vehkalahti et al., poor oral health have been shown to be a major factor in this kind of population study of the elderly [55]. Thus, taking into account that missing values and drop -out have poorer health, the present findings that differences in dental attendance were mainly related to presence or absence of natural teeth may only relate to healthy women.

## Conclusions

Most of the Norwegian elderly in our sample rated their oral health to be good and relatively few showed higher levels of psychological distress. Higher levels of psychological distress can negatively influence the elderly’s oral health, though other factors such as smoking and tooth loss are even stronger predictors of poor oral health. Dental care professionals should consider screening their elderly patients for psychological distress and customize the information about dental care and dental hygiene for this specific population.

## Abbreviations

HSCL-25: Hopkins Symptom Checklist-25
